# Structure of the human gonadotropin-releasing hormone receptor GnRH1R reveals an unusual ligand binding mode

**DOI:** 10.1038/s41467-020-19109-w

**Published:** 2020-10-20

**Authors:** Wei Yan, Lin Cheng, Wei Wang, Chao Wu, Xin Yang, Xiaozhe Du, Liang Ma, Shiqian Qi, Yuquan Wei, Zhiliang Lu, Shengyong Yang, Zhenhua Shao

**Affiliations:** 1 Division of Nephrology and Kidney Research Institute, State Key Laboratory of Biotherapy and Cancer Center, West China Hospital, Sichuan University, Chengdu, Sichuan 610041 China; 2 Department of Biological Sciences, Xi’an Jiaotong-Liverpool University, Suzhou, 215123 China; 3 Department of Urology, State Key Laboratory of Biotherapy, West China Hospital, Sichuan University and Collaborative Innovation Center for Biotherapy, Chengdu, 610041 China

**Keywords:** G protein-coupled receptors, X-ray crystallography

## Abstract

Gonadotrophin-releasing hormone (GnRH), also known as luteinizing hormone-releasing hormone, is the main regulator of the reproductive system, acting on gonadotropic cells by binding to the GnRH1 receptor (GnRH1R). The GnRH-GnRH1R system is a promising therapeutic target for maintaining reproductive function; to date, a number of ligands targeting GnRH1R for disease treatment are available on the market. Here, we report the crystal structure of GnRH1R bound to the small-molecule drug elagolix at 2.8 Å resolution. The structure reveals an interesting N-terminus that could co-occupy the enlarged orthosteric binding site together with elagolix. The unusual ligand binding mode was further investigated by structural analyses, functional assays and molecular docking studies. On the other hand, because of the unique characteristic of lacking a cytoplasmic C-terminal helix, GnRH1R exhibits different microswitch structural features from other class A GPCRs. In summary, this study provides insight into the ligand binding mode of GnRH1R and offers an atomic framework for rational drug design.

## Abstract (web summary)

The human gonadotropin-releasing hormone receptor GnRH1R is a GPCR with an important role in the human reproductive system and of interest as a drug target. Here, the authors present the 2.8 Å crystal structure of human GnRH1R with the high affinity antagonist drug Elagolix and the observed unusual ligand binding mode was further analysed with functional assays and molecular docking studies.

## Introduction

In humans, the hypothalamic–pituitary–gonadal (HPG) axis is critical for reproduction and the expression of sexual characteristics. The central regulator of the HPG axis is gonadotrophin-releasing hormone (GnRH), a decapeptide neurohormone produced by mammalian hypothalamus neurons^1,2^. The GnRH peptide can activate the GnRH receptor through the heterotrimeric G_q_ protein pathway. This in turn initiates the reproductive hormone cascade and releases gonadotropins: follicle-stimulating hormone and luteinizing hormone^3,4^. Alteration in the GnRH pulse pattern is observed in both physiological and pathological conditions associated with reproductive disorders^1^. There are two isoforms of the GnRH peptide in humans, GnRH-I and GnRH-II, and their effects are exerted by activation of the classical GnRH1 receptor (GnRH1R)^5,6^, which belongs to the rhodopsin G protein-coupled receptor (GPCR) family^7^. GnRH1R is expressed primarily on pituitary gonadotrope cells but also on lymphocytes and breast, ovary, and prostate cells^8–10^, and has emerged as a promising therapeutic target for the treatment of conditions including infertility^11^, uterine fibroids^12^, endometriosis^13^, and prostate cancer^14^.

In recent years, many GnRH-analog agonists have been developed for clinical applications that downregulate and desensitize the corresponding receptors on gonadotroph cells^15^, inhibiting the secretion of gonadotropins and sex steroids. Furthermore, GnRH-analog antagonists that can immediately block GnRH1R signaling have been synthesized^16^. Because of problems associated with peptide drugs, including poor stability and short half-life, orally active small-molecule drugs that act on GnRH1R are highly desirable and some have emerged from several chemical classes as potential avenues to treat reproductive hormone-related diseases^8,17^. The first nonpeptide GnRH1R antagonist was reported by Abbott Laboratories^18^. Since then, various scaffolds of nonpeptide antagonists have been designed and successfully advanced into different clinical trial phases^19^. For example, elagolix (NBI-56418), a derivative of uracil, was reported by Neurocrine Biosciences in the USA and was approved by the US Food and Drug Administration in 2018 for the treatment of moderate-to-severe pain associated with endometriosis. The drug is also under development for the treatment of uterine fibroids in women and prostate cancer and benign prostatic hyperplasia in men^20^. Takeda Company in Japan developed a series of thienopyridine derivatives, including sufugolix and relugolix, which have entered trials for endometriosis, prostate cancer, and uterine fibroids. In particular, relugolix was recently approved for marketing in Japan as a treatment for symptoms associated with uterine fibroids^21^.

Despite much recent progress in understanding the biochemical and pharmacological characteristics of GnRH1R, investigation of the molecular mechanisms of the interaction of the GnRH1 receptor with ligands still requires an experimental structure. In this study, we report the crystal structure of human GnRH1R in complex with the antagonist drug elagolix. Compared with other GPCRs, the GnRH1R structure contains an unusual orthosteric site in which the antagonist and the N terminus of the protein could co-occupy an enlarged binding pocket. Computational docking analyses and inositol phosphate (IP) accumulation assays of GnRH1R suggest different binding modes of agonist peptides and small-molecule antagonists with variable chemotypes.

## Results

### Crystallization of GnRH1R and overall structure

For lipidic cubic phase (LCP) crystallization, an initial GnRH1R construct was generated by replacing the third intracellular loop (ICL3; amino acids [aa] 243–256) with the thermostable *Pyrococcus abysi* glycogen synthase (PGS) domain^22^ (Supplementary Fig. 1a). To further improve the homogeneity of the receptor, the point mutation P128^3.39^K (Ballesteros–Weinstein numbering used in superscript) was incorporated into the engineered construct and the position 3.39 mutants were previously shown to make receptors stable in an inactive state, and were also reported in other recently elucidated GPCR structures^23,24^. Accordingly, GnRH1R with P128^3.39^K mutant retained a similar binding affinity with the endogenous ligand compared to wild-type GnRH1R (Supplementary Fig. 1b, c). However, we found that nonfusion GnRH1R with P128^3.39^K was unable to produce IP accumulation even under agonist stimulation (Supplementary Fig. 1e). Further gel filtration and differential scanning fluorimetry experiments were carried out for detergent-solubilized receptor, the melting temperature (*T*_m_ value) of elagolix-bound GnRH1R (P128^3.39^K)-PGS was 83 °C. These results revealed the antagonist elagolix combined with the mutant P128^3.39^K could confer GnRH1R with monodispersity and thermostability (Supplementary Fig. 2a–c). Finally, GnRH1R (P128^3.39^K)-PGS was crystallized using the LCP method in the presence of elagolix and the structure was successfully determined at 2.8 Å resolution (Supplementary Fig. 2d). Details of the data collection and structure refinement are provided in Supplementary Table 1.

**Fig. 1:**
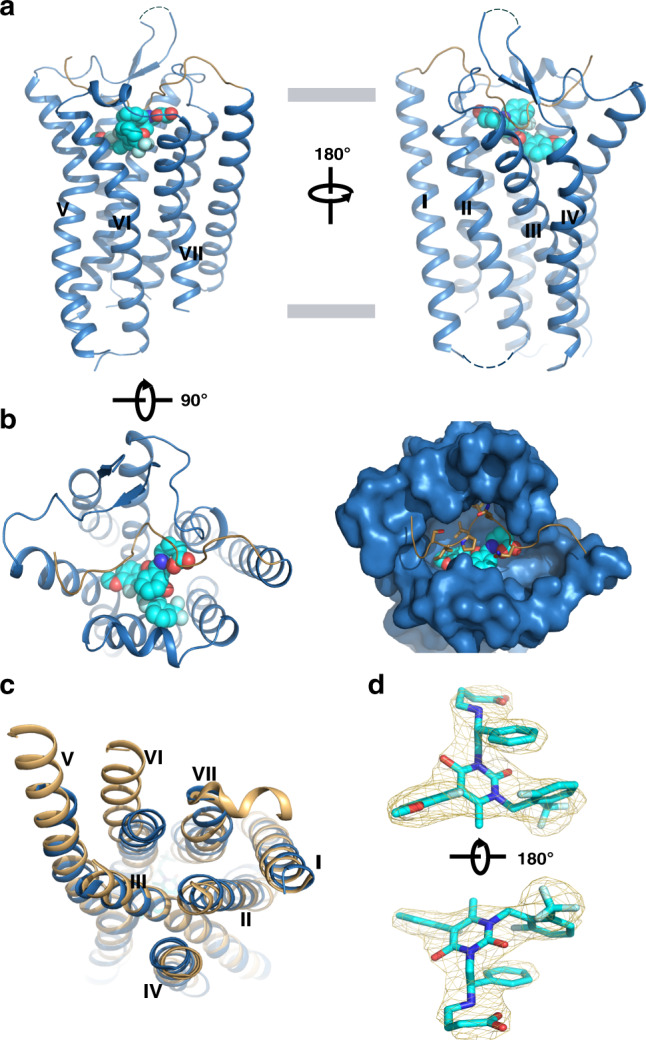
Overall structure of human GnRH1R in complex with antagonist elagolix. **a** View from within the plane of membrane. GnRH1R is shown in sky blue cartoon representation. The N terminus is displayed as sand cartoon. The antagonist elagolix is shown as sphere with cyan carbons. **b** View from the extracellular side of the membrane. Left one represents sky blue cartoon receptor with cyan sphere ligand. Right one shows the surface representation of 7-TM domain, key residues of the N terminus that are shown in sand sticks inserted the orthosteric pocket. The N terminus is displayed as sand cartoon. **c** View from the intracellular side of the membrane, structural comparison of GnRH1R (sky blue) with G protein -bound active NTS1R (6OS9, orange). **d** |Fo| − |Fc| omit map (contoured at 3.0*σ*) of the ligand elagolix. The antagonist elagolix is shown as cyan sticks.

GnRH1R was reported to belong to the *β*-branch class A GPCRs, which include orexin receptors, tachykinin receptors, and neurotensin receptors^25^. The overall architecture of full-length GnRH1R consists of seven canonical transmembrane (TM) helices but lacks a cytoplasmic C-terminal helix (helix 8) (Fig. 1a, b). Similar to peptide-activated GPCRs of this subfamily, the ECL2 of GnRH1R also forms an extended *β*-hairpin and is anchored to the extracellular tip of TM helix 3 (TM3) through a conserved disulfide bond between residues C114^3.25^ and C196 in ECL2. Interestingly, further structural comparison shows that GnRH1R displays a high root-mean-square deviation (RMSD) for the TM backbone Cα atoms (3.02 Å with OX2R (PDB ID: 4S0V), 2.34 Å with NK1R (PDB ID: 6E59), 3.11 Å with NPY1R (PDB ID: 5ZBH), 1.93 Å with ET_B_R (PDB ID: 5XPR), and 2.35 Å with NTS1R (PDB ID: 4BUO).

Compared with the structure of active NTS1R^26^, inward movements of TM5 and TM6 in GnRH1R from the intracellular side were observed, shrinking the cavity for G protein coupling (Fig. 1c). In addition, the typical conserved D^3.49^-R^3.50^-Y^3.51^ motif in most GPCRs is in fact the D^3.49^-R^3.50^-S^3.51^ motif in GnRH1R. An intrahelical salt bridge is observed between D138^3.49^ and R139^3.50^, as well as a polar interaction between R139^3.50^ and T265^6.33^, restricting the outward movement of those TMs involved in GPCR activation (Supplementary Fig. 3). In summary, the GnRH1R crystallized in complex with the antagonist drug elagolix represents an inactive conformation with respect to G protein coupling.

In contrast to the previously reported structures of *β*-branch class A GPCRs, one interesting feature of GnRH1R on the extracellular side is that the N-terminal region (aa 18–33) before TM1 is well folded and inserted into the orthosteric binding cavity with clear electron density in the structure (Supplementary Fig. 4a). This conformation of the N terminus in GnRH1R was not influenced by lattice packing contacts in the crystals (Supplementary Fig. 4b). Consistent with this observation, we performed a 200 ns molecular dynamics (MD) simulation of the GnRH1R structure in the presence and absence of elagolix, revealing that the N terminus displayed high stability over both MD simulation courses with low RMSD values (Supplementary Fig. 5).

### Structural basis of elagolix binding

In the GnRH1R-elagolix structure, elagolix adopts a conformation in which its uracil core undergoes intramolecular packing within phenyl rings at positions 1 and 3, as observed from unambiguous electron density in the orthosteric pocket (Fig. 1d and Supplementary Fig. 4c). The overall pocket in GnRH1R is defined by the N terminus, TM2, TM3, TM5, TM6, and TM7, forming a highly hydrophobic binding site. Only a few polar residues (D98^2.61^, N102^2.65^, K121^3.32^, and N305^7.35^) were found within 4 Å of the ligand.

**Fig. 2:**
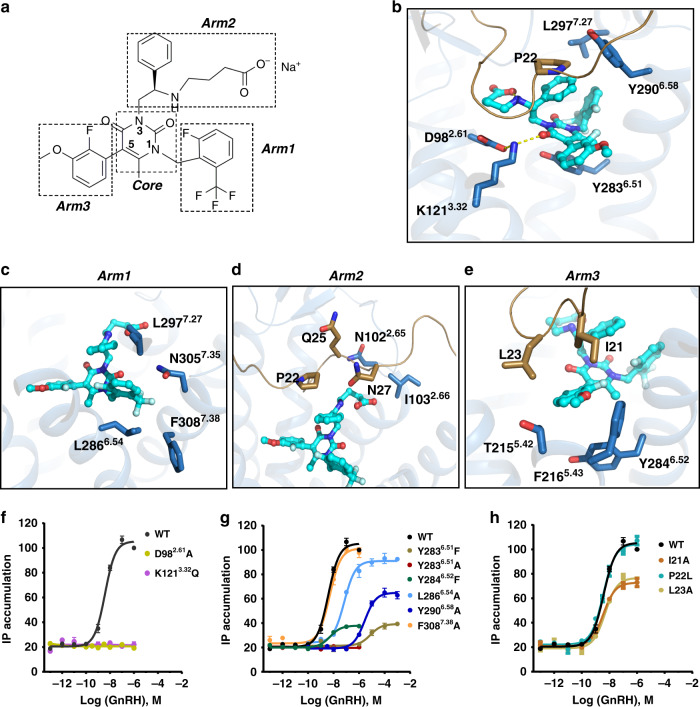
Interaction of elagolix with GnRH1R. **a** Two-dimensional structure of elagolix, the positions 1, 3, and 5 of elagolix uracil core are termed arm1, arm2, and arm3, respectively. **b**–**e** Key residues (sky blue sticks) involved in different parts of ligand binding are displayed in **b** (core), **c** (arm1), **d** (arm2), and **e** (arm3), respectively. The only hydrogen bonds net was shown as dotted yellow lines. **f** Agonist GnRH dose-dependent responses of GnRH1R wild-type (WT), D98^2.61^A, and K121^3.32^Q mutants by IP (inositol phosphate) accumulation assays. Both mutation abolished the GnRH-induced signaling. EC_50_ values are expressed as means ± SEM (*n* = 3) at three times independently experiment repeats with similar results. **g** Representative of effects of hydrophobic residues in orthosteric site on agonism of GnRH in IP accumulation assays. EC_50_ values are expressed as means ± SEM (*n* = 3) at three times independently experiment repeats with similar results. **h** Representative of effects of N-terminal residues of the receptor on agonism of GnRH in IP accumulation assays. HEK293T were transiently transfected with the wild-type or mutant receptors (without PGS), and IP accumulation was measured after stimulation with GnRH ligands for 2 h. EC_50_ values are expressed as means ± SEM (*n* = 3) at three times independently experiment repeats with similar results.

The detailed ligand-binding basis is presented in Fig. 2. The pyrimidine ring, as the core of the ligand elagolix, sits above the aromatic side chain of Y283^6.51^ and forms a hydrophobic *π*–*π* interaction with each other. However, the oxygen atom at position 4 is engaged in binding with the side chains of residues K121^3.32^ and D98^2.61^, forming only a polar interaction network in the elagolix binding site (Fig. 2a, b).

The benzyl group at position 1 (termed “arm1”) of the uracil core of elagolix extends towards the interface between TM6 and TM7, forming hydrophobic interactions with L286^6.54^, N305^7.35^, and L297 in the ECL3 region. In particular, its trifluoromethyl moiety projects into F308^7.38^ in TM7 (Fig. 2c). Position 3 of the uracil core is termed arm2, including a phenyl group along with a butyrate moiety. The phenyl group forms *π*–*π* interactions with residue P22 of the N terminus, whereas the butyrate moiety lies close to the top of the helix bundle, thus stacking with the main chains of residues Q25 and N27 in the N terminus via hydrophobic or van der Waals interactions and facing towards the extracellular site (Fig. 2d). Therefore, incorporation of the butyrate moiety in arm2 might increase the possibility of solvent access and water-mediated interactions may be included in the contacts between the ligand and receptor.

Arm3 (2-fluoro-3-methoxyphenyl at position 5 of uracil core) makes hydrophobic interactions with the side chains of residues I21 and L23 in the N terminus of the receptor, as well as T215^5.42^ and F216^5.43^ in TM5 (Fig. 2e). Interestingly, an extended hydrophobic cavity shaped by those residues is found in the arm3 recognition area in our current structure (Fig. 2e), which is consistent with previous structure–activity relationship studies, indicating that a large heteroaromatic ring substituent on arm3 is a promising candidate for GnRH1R^27^. The methyl group at position 6 of elagolix is suggested to be an additional critical moiety that contributes to high-affinity ligand binding^28^, probably by forcing arm3 ring into a different plane at approximately a 45° angle relative to the core plane, embedding arm3 into the hydrophobic pocket (Fig. 2e).

Among the interactions between the ligand and GnRH1R, it should be noted that the polar network residues composed of K121^3.32^-D98^2.61^ play critical roles by interacting with ligands. This observation is consistent with previous findings that these residues contribute to the binding of ligands, including agonists and antagonists^29^. Our functional assays showed that both K121^3.32^Q and D98^2.61^A mutations almost abolished the signaling response to agonist stimulation (Fig. 2f). In addition, to explore the effects of the hydrophobic environment in the binding pocket of GnRH1R, we generated several mutations in GnRH1R, for instance, Y283^6.51^F, L286^6.54^A, Y290^6.58^A, and F308^7.38^A. Among the mutations, the Y283^6.51^F replacement resulted in a significant reduction in IP accumulation (>1000-fold) compared to wild-type GnRH1R (Fig. 2g), which is consistent with a previous study showing that Y283^6.51^ was engaged in the ligand recognition and activation of GnRH1R^29^. More interestingly, the N terminus (I21, P22, and L23) of GnRH1R was observed to be involved in arm2 and arm3 binding of elagolix, and alanine replacements of three residues at the N terminus (I21, P22, and L23) all displayed a similar GnRH-induced response to that of wild-type GnRH1R (Fig. 2h). However, these mutants showed dramatically diminished inhibition of GnRH-induced IP accumulation (IC_50_) by elagolix (>1000-fold decrease for I21A, 988-fold for P22L, and 725-fold decrease for L23A) (Supplementary Fig. 6a and Supplementary Table 2), suggesting that the N terminus of GnRH1R potentially participated in ligand binding.

### Unusual binding pocket in GnRH1R

**Fig. 3:**
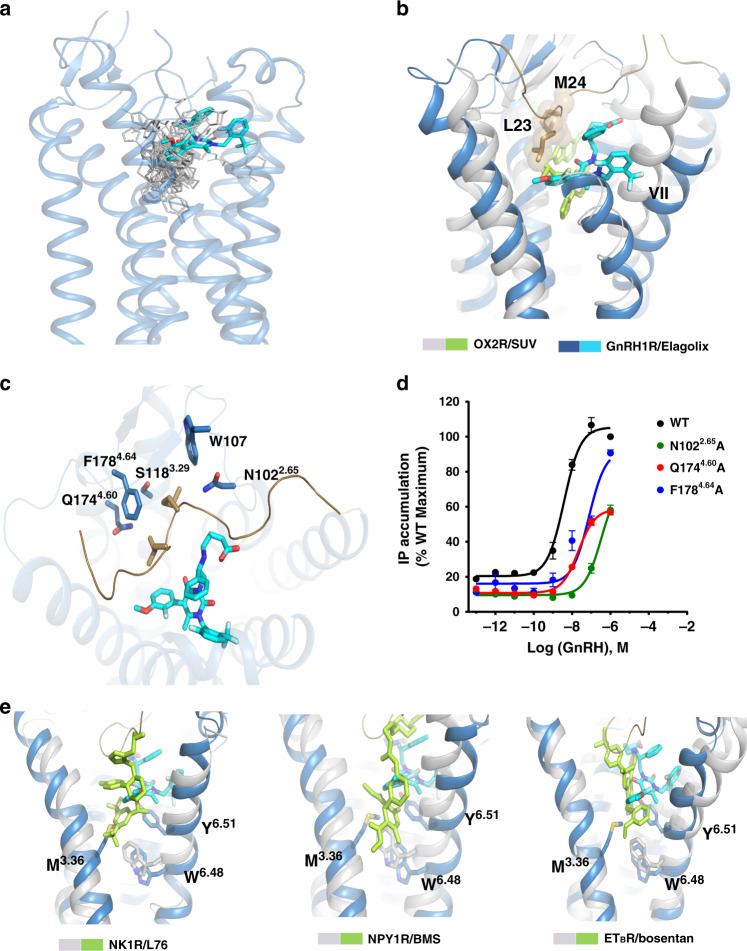
Unusual orthosteric pocket of GnRH1R. **a** Position comparison of elagolix in GnRH1R with small-molecule ligands in class A GPCRs family. The elagolix is shown as stick with cyan carbons, and the ligands from other GPCRs are shown as gray lines. **b** Structural superposition of GnRH1R with OX2R (gray cartoon, PDB ID: 4S0V), the ligand SUV (suvorexant) of OX2R is shown as green stick. In GnRH1R, two residues L23 and M24 (shown as cyan sticks with sphere) of the N terminus occupy the enlarged orthosteric pocket. **c** Key residues (sky blue sticks) within 4 Å of both L23 and M24 in GnRH1R. **d** Dose-dependent responses of GnRH1R WT and mutants (N102^2.65^A, Q174^4.60^A, and F178^4.64^A) to GnRH agonist determined by IP assays. See Supplementary Table 2 for detailed statistical evaluation. HEK293T were transiently transfected with the wild-type or mutant receptors (without PGS) and IP accumulation was measured after stimulation with GnRH ligands for 2 h. EC_50_ values are expressed as means ± SEM (*n* = 3) at three times independent experiment repeats with similar results. **e** Structural superposition of the elagolix-bound GnRH1R with L76 (L76075)-bound NK1R (PDB ID: 6E59), BMS (BMS-193885)-bound NPY1R (PDB ID: 5ZBH), and Bosentan-bound ET_B_R (PDB ID: 5XPR), respectively. Those three ligands (L76, BMS, and Bosentan) shown as green stick bound deeply in their corresponding receptor (shown as gray cartoon) orthosteric site. The toggle switch residue W^6.48^ is shown as a stick in all four receptors and both M^3.36^ and Y^6.51^ are shown in sky blue sticks forming the bottom wall of orthosteric pocket of GnRH1R.

In further comparison with elucidated rhodopsin GPCR family structures, the extracellular side of TM7 in GnRH1R showed an obvious 6 Å outward movement relative to that in the antagonist-bound OX2R structure, because the elagolix in GnRH1R is located closer to TM7 (Fig. 3a, b), resulting in an enlarged orthosteric pocket in the GnRH1R. The ligand elagolix appeared to occupy part of the pocket and a V-shaped N terminus of GnRH1R inserted into the binding pocket, which is similar to the N terminus of the CB1 receptor bound to antagonists^22,30^. However, it is worth noting that the N terminus of GnRH1R fits into a cavity that is defined by TM2, ECL1, TM3, and TM4, and is covered by the ECL2 region, in which some residues are topologically equivalently available in orexin receptors, ET_B_R and CXCR4 structures and were reported to mediate direct contact with their ligands^31,32^. Further careful inspection revealed that the N terminus overlapped with the ligands in those receptors (Supplementary Fig. 6b). In particular, some GPCRs were reported to have open orthosteric binding pockets and to be able to accommodate multiple ligands, including small molecules, peptides, or even proteins, so those ligands can occupy different regions of the binding pocket^33^. In the GnRH1R-elagolix complex structure, the two residues, L23 and M24, not only pack with the ligand in parallel but also contact the surrounding residues N102^2.65^, Q174^4.60^, and F178^4.64^ from TM2 and TM4, respectively (Fig. 3c). A previous study showed that the N terminus of GnRH1R had distinct roles in binding different ligands; it was not engaged in GnRH direct binding but participated in small-molecule NBI-42902 (elagolix analog) binding^29^. Together, our structure reveals an enlarged orthosteric pocket of GnRH1R that is occupied by both ligand and N-terminal regions.

In combination with our site-directed mutagenesis and cellular functional assays discussed above, alanine substitutions of four residues at the N terminus (I21, P22, L23, and M24) did not attenuate the GnRH-induced signaling response compared with that for the wild-type receptor (Fig. 2h and Supplementary Fig. 6a). However, almost complete loss of inhibition of the GnRH-induced response by elagolix was observed for the M24A mutant (Supplementary Fig. 6d and Supplementary Table 2), which is supported by a previous study showing that residue M24 was involved in elagolix analog binding but not native GnRH agonist binding^29^. These results suggest that the N terminus of GnRH1R might not be involved in agonist GnRH binding but participates in small-molecule antagonist binding, as is visible in our structure. Furthermore, we generated three mutations (N102^2.65^A, Q174^4.60^A, and F178^4.64^A) surrounding residues L23 and M24 in our structure. Interestingly, all three mutations caused notable decreases in IP accumulation, the expression level of N102^2.65^A and F178^4.64^A retained similar level with wild-type GnRH1R, whereas Q174^4.60^A displayed 88% expression level of wild type (Fig. 3d and Supplementary Table 2). Combined with previous reports that N102^2.65^, Q174^4.60^, and F178^4.64^ contribute to native GnRH binding^34,35^ (Supplementary Table 2), these findings may indicate an extended pocket in GnRH1R for peptide agonist binding.

Another notable difference between elagolix-bound GnRH1R and reported ligand-bound β-branch GPCR structures is the position of the ligands. The ligands in those β-branch GPCR structures extend deeply in the orthosteric pocket and make direct contact with W^6.48^ (Fig. 3e). The highly conserved residue W^6.48^ is reported as a “toggle switch” in most class A receptors, triggering the activation motion of the receptor^36^. However, according to our structural comparison, elagolix in the GnRH1 receptor seems to be close to the top of the classical pocket and directly contacts residue Y283^6.51^ (Fig. 3e), which but is located above the conserved residue W280^6.48^. Consequently, Y283^6.51^ together with Y284^6.52^ and M125^3.36^ are suggested to form the bottom wall of the orthosteric pocket in the antagonist-bound GnRH1R structure (Supplementary Fig. 7), decreasing the depth of the ligand-binding cavity and blocking access of the ligand to the toggle switch area. Our structure is consistent with a previous study showed by site-directed mutagenesis that residue W280^6.48^ was not involved in direct ligand binding^37^, suggesting that Y283^6.51^ might play an essential role in the GnRH1 activation process.

### Docking poses of different antagonists in GnRH1R

**Fig. 4:**
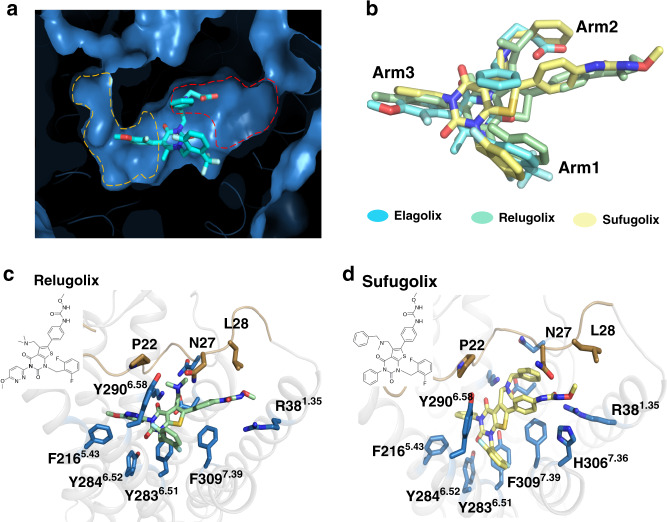
The feature of orthosteric pocket in GnRH1R and predictive docking pose of small-molecule antagonists. **a** Cutaway view of the elagolix binding pocket in GnRH1R, more cavities related with arm2 and arm3 are circled by yellow and red color, respectively. **b** Ligands superposition in GnRH1R-elagolix, relugolix, and sufugolix are shown in cyan, pale cyan, and pale yellow sticks, respectively. **c** Relugolix and **d** sufugolix binding mode of GnRH1R, key residues involved in potential interaction are represented as sky blue sticks.

The structure we obtained reveals the shallow nonpeptide antagonist binding site and the basis of GnRH1 receptor recognition of a ligand. As seen in our structure, arm2 in elagolix points to the extracellular surface of the receptor, which is solvent accessible. This large cavity allows us to suggest more recognition mechanisms of GnRH1R with extended groups of arm2 than in different chemotypes (Fig. 4a).

To explore the binding mechanism of GnRH1R with nonpeptide antagonists, we carried out computational docking of the clinically approved drug relugolix and its analog sufugolix into our structure. Obviously, these three ligands have differences in the arm2 regions (Fig. 4b). For each ligand, the top-ranked pose was selected and further refined by 200 ns of MD simulation. The docking pose of the thienopyrimidine core group of relugolix or sufugolix overlaps with that of the uracil core of elagolix, as did arm1 and arm3 (Fig. 4b). However, arm2 in both relugolix and sufugolix has a longer methoxyphenyl-urea moiety than that in elagolix and occupies a larger cavity than arm2 in elagolix (Figs. 4c, d). In particular, conformational rearrangement of the side chains of residues R38 and H306 was observed in the docked structures; they formed strong hydrogen bonds with the methoxyphenyl-urea moiety of arm2 in relugolix, forming an extended cavity for ligand binding (Supplementary Fig. 6c). This observation is consistent with a previous study showing that residues R38^1.35^ and H306^7.36^ contribute directly to ligand binding^29^ (Supplementary Table 2).

More interestingly, in the simulations, we found that the N terminus and ECL1 regions in the docked sufugolix-bound GnRH1R structure swung away from those in the elagolix-bound structure and the extracellular region of TM7 made outward movements (Supplementary Fig. 6c), making more space to bind with extended arm2 in sufugolix. These results suggest the plasticity of the orthosteric binding pocket of GnRH1R with respect to different ligands and provide the possibility to design orally deliverable small molecules with activity towards the receptor owing to the solvent-accessible channel in GnRH1R.

### Structural features of microswitches in human GnRH1R

Most GPCRs are reported to transduce signals into intracellular effectors as a result of conformational rearrangements of common microswitches^38,39^. Considering the unusual ligand recognition characteristics and absence of the cytoplasmic C-terminal helix of GnRH1R as we described above, we sought to provide more insight into the structural features of microswitches in GnRH1R, including the CW^6.48^xP^6.50^ motif, P^5.50^-I^3.40^-F^6.44^ motif, NP^7.50^xxY^7.53^ motif, and D-R^3.50^-Y motif.

**Fig. 5:**
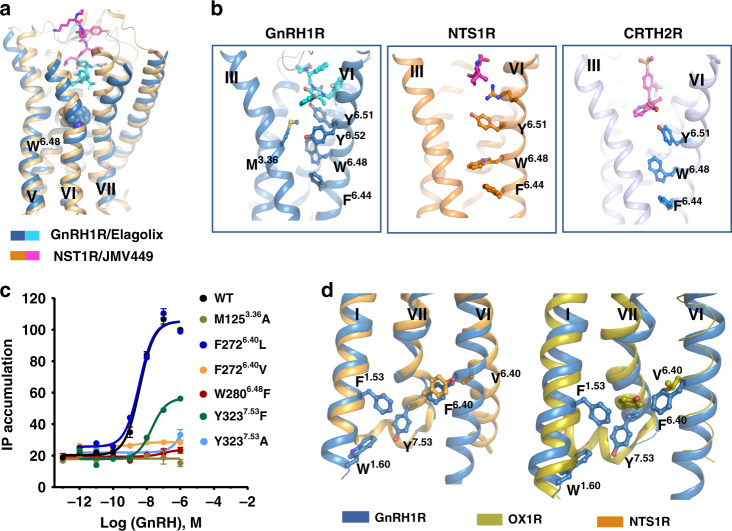
The structural features of microswitches in GnRH1R. **a** Structural superposition of GnRH1R with active NTS1R (PDB ID: 6OS9, orange cartoon). Agonist JMV449 of NTS1R is shown magentas. Both elagolix and JMV449 bound to a shallow pocket that is located near the extracellular region of those two receptors. **b** The side chains of the hydrophobic Y^6.51^-W^6.48^-F^6.44^ motif in TM6 are shown as sticks in GnRH1R (left), NTS1R (middle, PDB ID: 4S0V), and CRTH2R (right, PDB ID: 6D26), respectively. **c** Effects of GnRH agonist on activating GnRH1R WT, M125^3.36^A, F272^6.40^L, F272^6.40^V, W280^6.48^F, Y323^7.53^F, and Y323^7.53^A mutants expressed in HEK293T cells independently, monitored by IP1 accumulation assays. HEK293T were transiently transfected with the wild-type or mutant receptors (without PGS) and IP accumulation was measured after stimulation with GnRH ligands for 2 h. EC_50_ values are expressed as means ± SEM (*n* = 3) at three times independently experiment repeats with similar results. **d** The rotamer comparison of NP^7.50^xxY^7.53^ motif of GnRH1R with inactive OX2R (PDB ID: 4S0V, green cartoon) and active NTS1R (PDB ID: 6OS9, orange cartoon) reveals an unusual conformation of Y^7.53^ in GnRH1R. The side chain of F^1.53^, W^1.60^, and F^6.40^ residues are shown as sticks with corresponding color to their receptors.

Compared with the NTS1R structure, GnRH1R reveals a similar shallow ligand-binding site that is located near the extracellular region (Fig. 5a). In particular, residue Y^6.51^ in both GnRH1R and NTS1R connects the ligand via direct contacts and engages in hydrophobic stacking interactions with residue W^6.48^ (Fig. 5b), forming hydrophobic motif Y^6.51^-W^6.48^-F^6.44^ in both receptors. A similar motif (Y/F^6.51^-W^6.48^-F^6.44^) was also found in some class A GPCRs, such as CRTH2R (Fig. 5b). Unlike some GPCRs in which ligands could contact residue W^6.48^ directly, which functions as a toggle switch in receptor activation, in GnRH1R, the special motif Y^6.51^-W^6.48^-F^6.44^ together with Y^6.52^ in TM6 is suggested to be a critical structural motif involved in mediating the propagation of signal transmission. Our IP accumulation results indeed revealed that Y283^6.51^ and Y284^6.52^ greatly contribute to GnRH1R activation (Fig. 2g); moreover, the W280^6.48^F mutation almost abolished the signaling response (Fig. 5c), which is consistent with previous mutagenesis data showing that residues Y283^6.51^ and Y284^6.52^ are crucial for ligand binding^29^.

The P^5.50^-I^3.40^-F^6.44^ motif is conserved in most GPCRs and characterized as a key connector that can facilitate the movements of the cytoplasmic region of TM6. In GnRH1R, the typical residue I^3.40^ is substituted by a simple side chain A129^3.40^ and structural superimposition between GnRH1R and β2 receptors^40,41^ reveals a special rotamer conformation of F276^6.44^ in the antagonist-bound GnRH1R structure (Supplementary Fig. 8a). On the other hand, residue Y^5.58^ is important in receptor activation, with its side chain pointing towards the interface between TM3 and TM6; only 4% of class A receptors, including GnRH1R, have asparagine at this 5.58 position, which is implicated in a polar interaction with S136^3.47^ in GnRH1R, thus leading to TM6 packing tightly with TM3 and TM5 in GnRH1R. Coincidentally, N^5.58^ in NTS1R was reported to make a great contribution to the activation process and replacement of residue N231^5.58^ by tyrosine abolished the GnRH-dependent signaling of GnRH1R (Supplementary Fig. 8b and Supplementary Table 2).

In most GPCRs, residue Y^7.53^ in the N^7.49^P^7.50^xxY^7.53^ motif is located at the cytoplasmic tip of TM7 and is followed by helix 8. Residue Y^7.53^ displayed rotamer rearrangement upon activation, pointing to the interior of TM domains in activated GPCRs. Obviously, in GnRH1R, Y323^7.53^ in the D^7.49^-P^7.50^xxY^7.53^ motif contacted F56^1.53^ and W63^1.60^ in TM1, displaying a different conformation from tailed antagonist-bound GPCRs because of the absence of the helix 8 tail. However, Y323^7.53^ in elagolix-bound GnRH1R exhibits a stable conformation over our 200 ns MD simulation (Supplementary Fig. 8c). Our functional assays indicated that the replacement Y323^7.53^A in GnRH1R abolished activation, whereas Y323^7.53^F retained the partial ability to simulate GnRH-induced signaling compared to wild-type GnRH1R (Fig. 5c and Supplementary Table 2). In addition, it is worth noting that residue F272^6.40^, which is highly conserved in tailless mammalian GnRH1R, is substituted by short hydrophobic residues in most GPCRs (Supplementary Fig. 8d). Consistently, our data show that the substitution F272^6.40^L retained the same level as the activation of wild-type GnRH1R, whereas the substitution F272^6.40^V nearly abolished the activation of the receptor (Fig. 5c and Supplementary Table 2). Function of the residue F272^6.40^ in activation process should be further explored in active structure of GnRH1R. Together, our results show that F272^6.40^ and Y323^7.53^ were important for GnRH1R activation, even in the absence of helix 8.

## Discussion

The residue D^2.50^ is highly conserved across all class A GPCRs and was reported to be involved in either allosteric sodium binding or activation; however, in GnRH1R, D^2.50^ is not present but is replaced by N^2.50^ at the same position. Thus, the N^2.50^-D^7.49^ pair becomes one of the features of GnRH1R that forms direct polar interactions in the GnRH1R structure (Supplementary Fig. 8e). To understand the role of the special N^2.50^-D^7.49^ pair in GnRH1R, we performed IP accumulation assays for GnRH1R mutants N^2.50^D and D^7.49^N, as well as the reversed double mutant N^2.50^D/D^7.49^N. The results show that all the mutants almost abolished GnRH-induced signaling (Supplementary Fig. 8b), which is consistent with a previous study^42^. Water molecules were investigated to stabilize the GPCR structure and mediate the transition of different GPCR conformation states by rearranging the conserved hydrophilic network forming conserved amino acids in different helices^43^. In addition, residue Y^7.53^ was reported to be involved in the water-mediated network with the surrounding residues^43^. In GnRH1R, when we replaced residue Y323^7.53^ with alanine or phenylalanine, respectively, they all decreased the activity potency of the receptor. In combination with the critical role of the N^2.50^-D^7.49^ pair in GnRH1R, we suggest that the N^2.50^-D^7.49^ pair and Y323^7.53^ should be critical for GnRH1R activation and might be involved in the interhelical-mediated network.

The N terminus of GnRH1R inserts into the orthosteric site but may not contribute to receptor-G_q_ signaling activation. The N termini of GPCRs have been reported to undergo structural rearrangement when the receptor binds to an agonist^44,45^. Therefore, structures of GnRH1R in complex with representative agonists would aid better understanding of the properties of the N terminus of the receptor, as well as the structural features of microswitches. In addition, GnRH1Rs are reported to be expressed in different tissues and display variable G protein signaling, resulting in complex pharmacology and physiology. Therefore, structural study of the receptor in complex with signaling effectors will be pursued to understand the detailed mechanisms of this receptor, which will facilitate new drug development for HPG axis regulation via GnRH1R.

In conclusion, the GnRH-GnRH1R system is of outstanding importance for basic biology and endocrinology, and GnRH1R is a promising drug target for the treatment of sex hormone-dependent diseases. Our results reveal the atomic details of GnRH1R and provide insight into the molecular mechanism of small-molecule ligand interactions with this receptor. By structural comparison and previous mutagenesis data^29,35^, the results reveal an unusual orthosteric binding pocket in GnRH1R, in which the small-molecule antagonist elagolix and several N-terminal residues pack together and co-occupy the site. In addition, in combination with our computational docking results, two critical networks are confirmed in GnRH1R: a polar interaction network formed by residues D98^2.61^ and K121^3.32^ is a determinant for GnRH1R with ligand recognition, whereas the hydrophobic Y^6.51^-Y^6.52^-W^6.48^-F^6.44^ motif in TM6 of GnRH1R is critical for signal transmission upon extracellular stimuli.

## Methods

### Cloning, expression, and purification

The nucleotides sequence of human codon-optimized GnRH1 receptor (GnRH1R; Supplementary Table 3) was cloned into a modified pFastbac1 (Invitrogen) vector with a hemagglutinin (HA) signal sequence followed by a Flag tag at the N terminus and a 10× his tag at the C terminus. To facilitate GnRH1R crystallization, a synthetic DNA sequence for translating the 196 residues PGS (PDB ID: 2FBW) was inserted into the ICL3 (residues 243–256). Finally, a mutation GnRH1R P128^3.39^K was introduced into the construct to improve the receptor thermostability.

The final construct GnRH1R (P128^3.39^K)-PGS was transformed into DH10Bac^™^ *Escherichia coli* for transposition and then recombinant bacmid transfected into Sf9 insect cells to produce high-titer recombinant baculovirus (>10^8^ viral particles per ml) using Bac-to-Bac Baculovirus Expression System (Invitrogen). The Sf9 cells were infected with recombinant baculovirus at a cell density of 2.5 × 10^6^/ml and cultured at 27 °C for 48 h with antagonist elagolix (Selleck) at the final concentration of 1 μM in the medium. Cells were collected by centrifugation at 8000 × *g* for 10 min at 4 °C and stored at −80 °C.

Sf9 cell membranes were lysed by thawing frozen cell pellets in a hypotonic buffer containing 10 mM HEPES pH 7.5, 20 mM KCl, 10 mM MgCl_2_, 160 μg ml^−1^ benzamidine, and 100 μg ml^−1^ leupeptin, then centrifuged at 10,000 × *g* for 30 min at 4 °C; the procedure was repeated three times. The membranes were further washed with a high osmotic buffer containing 10 mM HEPES pH 7.5, 20 mM KCl, 10 mM MgCl_2_, 1 M NaCl, 160 μg ml^−1^ benzamidine, and 100 μg ml^−1^ leupeptin three times to remove the soluble proteins. The washed membranes were then resuspended in the hypotonic buffer and incubated at 4 °C for 2 h in the presence of 50 μM elagolix. The membranes were then solubilized in a buffer containing 50 mM HEPES pH 7.5, 500 mM NaCl, 0.5% (w/v) Lauryl maltose-neopentyl glycol (LMNG, Anatrace), 0.1% sodium cholate, 0.1% cholesteryl hemi-succinate (CHS), 10% (v/v) glycerol, 160 μg ml^−1^ benzamidine, 100 μg ml^−1^ leupeptin, and 2 mg ml^−1^ iodoacetamide for 2.5 h at 4 °C. The supernatant was isolated by centrifugation at 125,000 × *g* for 30 min, supplemented with a final concentration of 10 mM imidazole, and incubated with TALON IMAC resin (TaKaRa) overnight at 4 °C. After binding, the resin was collected by centrifugation at 500 × *g* for 5 min, resuspended with wash buffer (50 mM HEPES pH 7.5, 500 mM NaCl, 0.025% LMNG, 0.005% sodium cholate, 0.005% CHS, 5% (v/v) glycerol) supplemented with 160 μg ml^−1^ benzamidine, 100 μg ml^−1^ leupeptin, 10 mM imidazole, and 1 μM elagolix, and was repeated one more time. The resin was loaded onto a glass column and washed with ten column volumes of wash buffer supplemented with 160 μg ml^−1^ benzamidine, 100 μg ml^−1^ leupeptin, 20 mM imidazole, and 1 μM elagolix. The GnRH1R protein was eluted with 10 ml elute buffer containing 50 mM HEPES pH 7.5, 500 mM NaCl, 0.025% LMNG, 0.005% sodium cholate, 0.005% CHS, 5% (v/v) glycerol, 160 μg ml^−1^ benzamidine, 100 μg ml^−1^ leupeptin, 200 mM imidazole, 10 μM elagolix, and then treated with PNGase F to remove galactosylated modification for 5 h at 4 °C. Finally, the concentrated protein was loaded onto a Superdex 200 10/300 increase size exclusion column (GE Healthcare) with a buffer containing 25 mM HEPES pH 7.5, 150 mM NaCl, 0.025% LMNG, 0.005% sodium cholate, 0.005% CHS, and 1 μM elagolix.

### LCP crystallization

For crystallization, the purified GnRH1R-elagolix complex was concentrated to 35 mg ml^−1^ and crystallized using the LCP method^46^. The sample of complex was mixed with the lipid (monoolein and cholesterol 10 : 1 by mass) at weight ratio of 2:3 using a syringe mixing apparatus at room temperature^47^. The mesophase was then dispensed onto glass sandwich plates in 40 nl drops and overlaid with 800 nl precipitant solution using a Gryphon LCP robot (Art Robbins Instruments). Crystals appeared after 1 day and grew to full size after 1 week at 20 °C in the following overlay precipitant condition: 100 mM sodium cacodylate pH 6.0–6.5, 30–38% PEG400, and 150–350 mM ammonium nitrate (Supplementary Fig. 2d). The crystals were collected from LCP matrix using MiTeGen loops and cryoprotected in liquid nitrogen immediately.

### Data collection, structure determination, and refinement

X-ray diffraction data were collected at beamline 41XU at the Spring-8, Hyogo, Japan, using a beam size of 10 μm and a Pilatus3 6 M detector (X-ray wavelength 1.0000 Å). A raster system was used to find the best-diffracting parts of crystals. For each crystal, 20 images were collected with 0.5° oscillation and 1 s exposure without attenuation of the beam. Full datasets of elagolix-bound GnRH1R (P128^3.39^K)-PGS complex were assembled from 23 crystals owing to the radiation damage of crystals. Data were indexed, integrated, scaled, and merged by using HKL300^48^. The dataset was processed in space group P3_1_21 and a resolution of 2.80 Å was selected according the *CC*_1/2_ criterion.

The structure of elagolix-bound GnRH1R (P128^3.39^K)-PGS complex was solved by molecular replacement with Phaser^49^ using human OX2R^31^ (PDB ID: 4S0V) and PGS^50^ (PDB ID: 2BFW) as independent search models. The solution was improved through iterations of manual building in Coot^51^, followed by refinement using Refmac5^52^ in the CCP4 package and Phenix^49^. Refinement parameters for elagolix ligand were generated using PRODRG^53^ web server. Statistics for data collection and refinement are included in Supplementary Table 1. The final structure had 93.1% of residues in the favored region of the Ramachandran plot, 6.9% in the allowed region, and 0 residues disallowed. Figures were prepared using Pymol (Schrodinger LLC).

### Receptor binding assay

The COS-7 cells were cultured in 96-well black view cell culture plates and transiently expressing GnRH1R (UniProt accession P30968) wild-type and the P128^3.39^K mutant receptors using Fugene HD transfection reagent according to the manufacturer’s menu. After 48 h incubation, culture medium was then aspirated and replaced with 100 μl 0.1% BSA-HEPES (10 mM)-buffered opti-media (phenol red-free) with a series of increasing concentration of Cy5-GnRH (Chinese peptides, Hangzhou) in the absence (total binding) or presence of 100-fold higher concentration of cold-ligand, GnRH peptide (non-specific binding). The assay was conducted in triplicate. The cells were then incubated at 4 °C for 4 h. Cells were washed twice using cold Dulbecco’s phosphate-buffered saline (DPBS) to remove unbound ligand. Fluorescent intensity was measured on the PHERAstar FS using the 640 nm excitation/680 nm emission optic module.

### Differential scanning fluorimetry

Protein samples were purified with antagonist elagolix. The concentrated protein was diluted to 2 μM in the 25 μl standard assay condition with the buffer containing 25 mM HEPES pH 7.5, 150 mM NaCl, 0.05% DDM, 0.01% CHS. The antagonist elagolix was incubated with receptor for 20 min before adding the BODIPY FL-l-cystine dye (Invitrogen), which was at 2 μM final concentration. The incubation was performed on the ice with the elagolix at final concentration of 10 μM. The scanning was performed at 0.5 °C temperature intervals from 4 °C to 90 °C by setting the FAM filter (450–490 nm excitation, 515–530 nm emission).

### IP accumulation assays

The cDNA of GnRH1R (UniProt accession P30968) wild-type was sub-cloned into pcDNA3.1 (+) expression vector with a HA signal sequence followed by a Flag tag at the N terminus and expressed in HEK293T cells. Point mutations (Supplementary Table 4) used in our study were generated by using Q5 site-Directed Mutagenesis kit (NEB). HEK293T cells were cultured in a six-well plate with Dulbecco’s modified Eagle’s medium supplemented with 10% (v/v) fetal bovine serum at a density of 3 × 10^5^ cells per well. Cells were collected 48 h post transfection. Cell surface expression of GnRH1R mutants were measured by Flow Cytometry using ANTI-FLAG M2-FITC antibody (Sigma, F4049).

IP accumulation was determined using Cisbio IP1 assay kit according to the manufacturer′s instructions. For agonist GnRH peptide stimuli, in brief, the harvested cells were distributed in a low volume 96-well dish by adding increased concentration of agonist GnRH peptide for 2 h. For antagonist curves, cells were pre-treated with elagolix (10^−4^–10^−12^ M) for 1 h, and then stimulated with optimized concentration of GnRH peptide for 2 h. IP1 was quantified using BIOTEK Cytation3 reader with excitation at 320 nm, emission at 620 nm and 665 nm. The accumulation of IP1 was calculated according to a standard dose–response curve in GraphPad Prism 7 (GraphPad Software). Data were represented as the mean ± SEM from three independent experiments and all experiments were repeated at least three times.

### Molecular docking

The fusion protein from the GnRH1R crystal structure was removed, the P128^3.39^ K mutation was restored to wild type, and the missing regions of ECL2 and ICLs were modeled ab initio using MODELLER v9.14102^54^ to obtain a wild-type receptor structure. After preparing GnRH1R with standard preparation procedures, molecular docking study was performed to determine the putative docking of GnRH1R antagonist (relugolix or sufugolix) with GnRH1R structure using the CDOCKER program^55^ in the BIOVIA Discovery Studio 3.1 (Accelrys, Inc., San Diego, CA, USA). Atomic coordinates for relugolix and sufugolix were optimized in the level of ωB97X/def2-TZVP using ORCA 4.0 software^56^. The highest scoring conformation was selected for further MD analysis.

### MD simulations

The MD simulations were conducted on the basis of the crystal structure of antagonist elagolix-bound GnRH1R. PGS fusion protein and ligand were removed from the structure and the P128^3.39^K mutation was restored to wild type. The missing regions of ECL2 and ICLs were further modeled ab initio using MODELLER v9.14102^54^. All-atom MD simulations were performed to further equilibrium the whole GnRH1R antagonist system and to refine receptor–ligand interactions. Parameters for the MD simulations were generated using the CHARMM-GUI web interface^57–59^ using the CHARMM36 forcefield^60^ with CGenFF^61^ for ligands. The disulfide bridge between C114 and C196 of the receptor was maintained. Hydrogen atoms were added at physiological pH (7.0) with the PROPKA^62^ tool. An 18 Å buffer of TIP3P waters, 83 × 83 Å^2^ homogeneous membranes composed of POPC, and 0.15 M (NaCl) salinity were used to reproduce physiological conditions. System without antagonist (apo state) was also analyzed for control purposes. The Particle-mesh Ewald method was used to treat the long-range electrostatic interactions, using a cut-off distance of 12 Å. The resulting system was geometry-optimized and then equilibrated for 10 ns using restraints on the GnRH1R backbone and ligand heavy atoms followed by a 200 ns production run. All MD simulations were performed with GROMACS simulation package.

### Reporting summary

Further information on research design is available in the Nature Research Reporting Summary linked to this article.

## Supplementary information

Supplementary Information

Peer Review File

Reporting summary

## Data Availability

Coordinates and structure factors for the antagonist elagolix-bound GnRH1R128^3.39^K-PGS complex are deposited in the Protein Data Bank under accession code PDB ID: 7BR3 (10.2210/pdb7BR3/pdb). The PDB codes referenced in this paper 2BFW for PGS domain and for OX2R as a molecular replacement model. Other data generated or analyzed during this study are included in this published article (and its Supplementary Information) or are available from the corresponding author on reasonable request. Source data are provided with this paper.

## Source data

Source Data
