# Supplementary material for: Structure of the human gonadotropin-releasing hormone receptor GnRH1R reveals an unusual ligand binding mode
Source: Nat Commun. 2020 Oct 20;11:5287. doi: 10.1038/s41467-020-19109-w (PMC7576152; doi:10.1038/s41467-020-19109-w)
Supplement: Supplementary file 1 — Supplementary Information [file 41467_2020_19109_MOESM1_ESM.pdf]

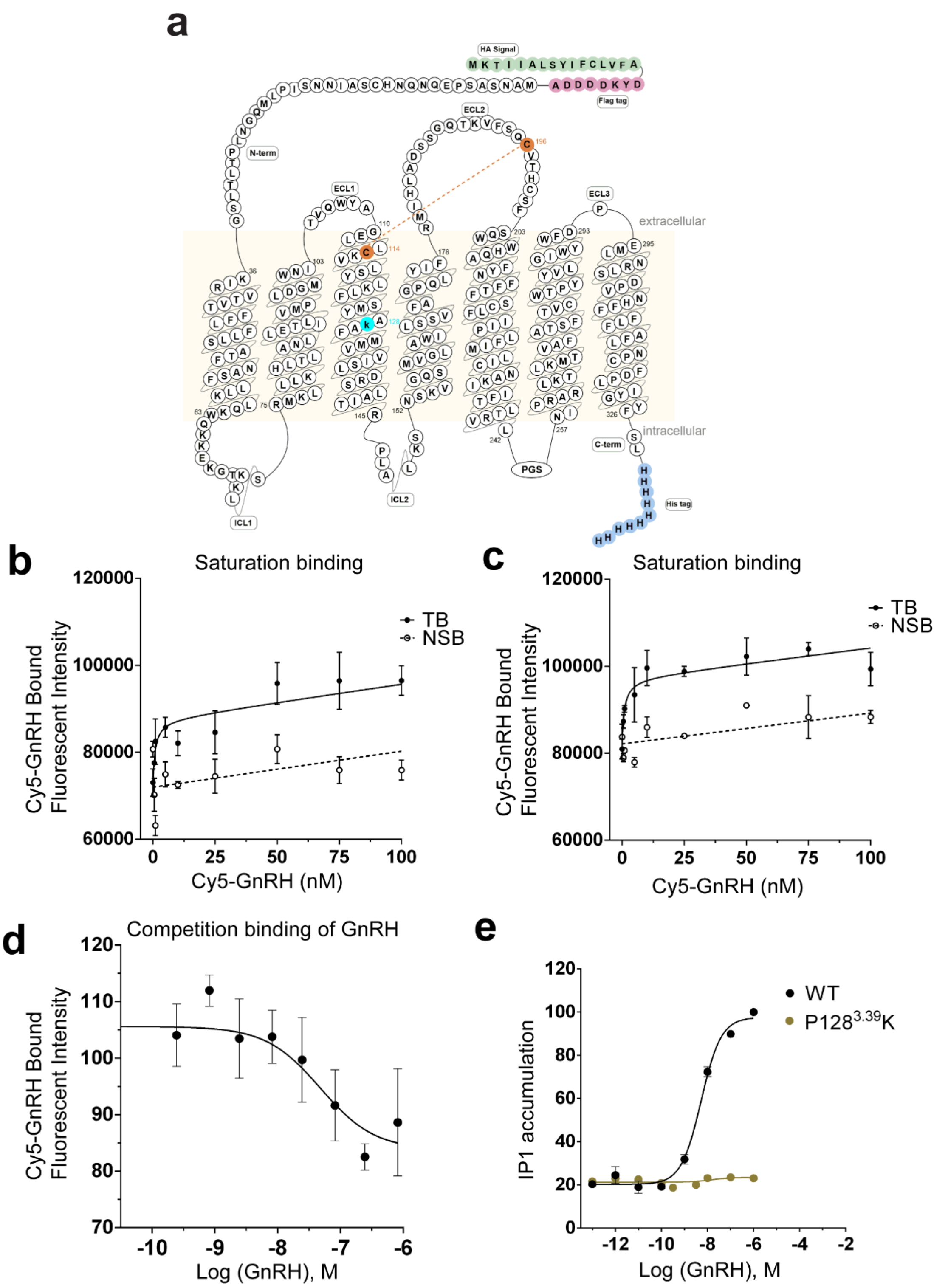

Supplementary Figure 1. GnRH binding assay and IP accumulation assay of GnRH1R constructs.

(a) Snake plot of the GnRH1R construct for crystallization. Residues 243-256 of ICL3 were replaced by PGS fusion domain. Mutation P128$^{3.39}$K is highlighted in cyan color. The conserved disulfide bond is indicated by a dashed orange line. HA signal, Flag

tag and His tag are shown in green, salmon, and violet respectively.

(b) Saturation binding of Cy5-GnRH on the wild-type (WT) GnRH1R. Fluorescent intensity was measured at Ex640/Em680 nm. The total binding (TB) and non-specific binding (NSB) were determined in the absence or presence of a 100-fold higher concentration of GnRH respectively. The K_d value is about 0.82 ± 1.4 nM. The *K*d values are expressed as means ± SEM (n=3) at 2 times independently experiment repeats with similar results.

(c) Saturation binding of Cy5-GnRH on the GnRH1R with P128$^{3.39}$K mutation. This mutant still retain the same level affinity (K_d =0.92 ± 1.3 nM) with GnRH as the WT GnRH1R. The *K*d values are expressed as means ± SEM (n=3) at 2 times independently experiment repeats with similar results.

(d) Competition binding of the agonist GnRH to the GnRH1R with P128$^{3.39}$K mutation. The pIC_{50} value is 7.3 ± 0.72. For experiments (b), (c), (d), COS-7 cells were transiently transfected with the wild-type or P128$^{3.39}$K mutant receptors (without PGS), and Fluorescent intensity was measured after stimulation with Cy5-GnRH ligands for 4 h. The pIC_{50} values are expressed as means ± SEM (n=3) at 2 times independently experiment repeats with similar results.

(e) IP accumulation assay of GnRH1R WT and GnRH1R P128$^{3.39}$K mutant. HEK293T were transiently transfected with the wild-type or P128$^{3.39}$K mutant receptors (without PGS), and IP accumulation was measured after stimulation with GnRH ligands for 2 h. EC_{50} values are expressed as means ± SEM (n=3) at 3 times independently experiment repeats with similar results. The mean EC_{50}± SEM (nM) are shown in Supplementary Table 2.

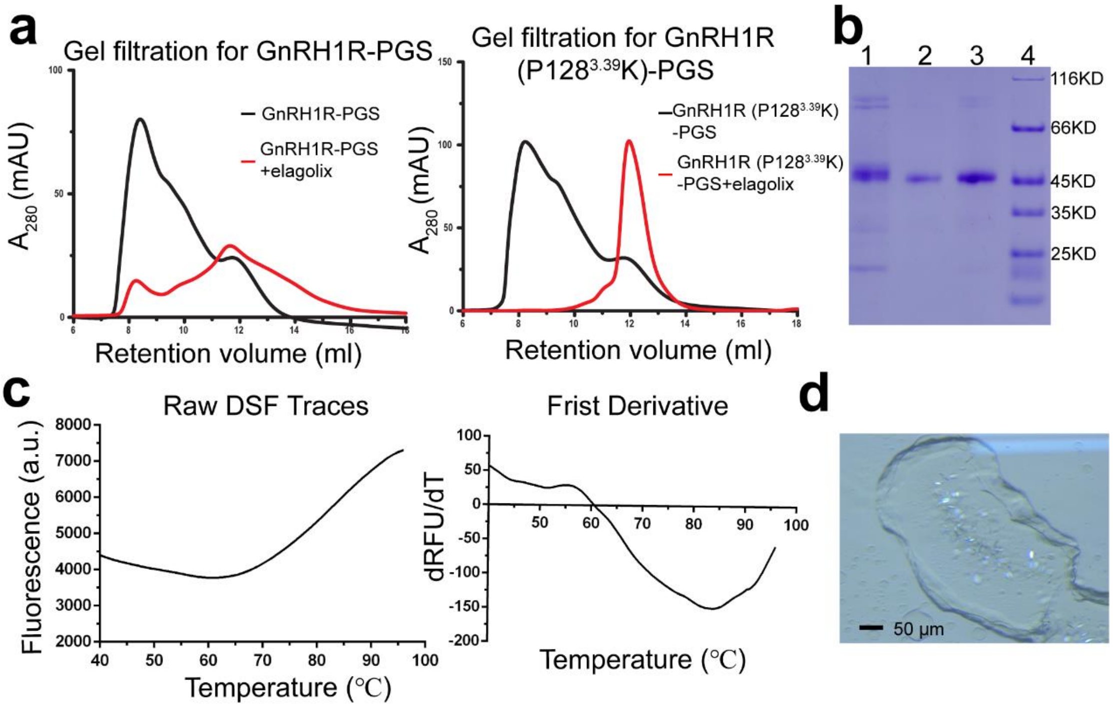

Supplementary Figure 2. Purification of the GnRH1R constructs.

(a) Superdex 200 gel filtration traces for both GnRH1R-PGS and GnRH1R ($P128^{3.39}K$)-PGS in the absence or presence of GnRH1R antagonist elagolix.

(b) SDS-PAGE analysis of samples in different steps of elagolix bound GnRH1R ($P128^{3.39}K$)-PGS purification. 4 lanes from left to right, 1: TALON IMAC chromatography purified receptor, 2: the deglycosylaed receptor with PNGase F, 3: final sample after gel filtration, 4: Marker (MW in KD at right).

(c) Raw DSF trace of the elagolix-bound GnRH1R ($P128^{3.39}K$)-PGS and first derivative analysis of data in Raw DSF trace. The apparent melting temperature (T_m value = 83°C) revealed a thermostabilized receptor for crystallization.

(d) Polarizing microscopy image of GnRH1R crystals in LCP drop.

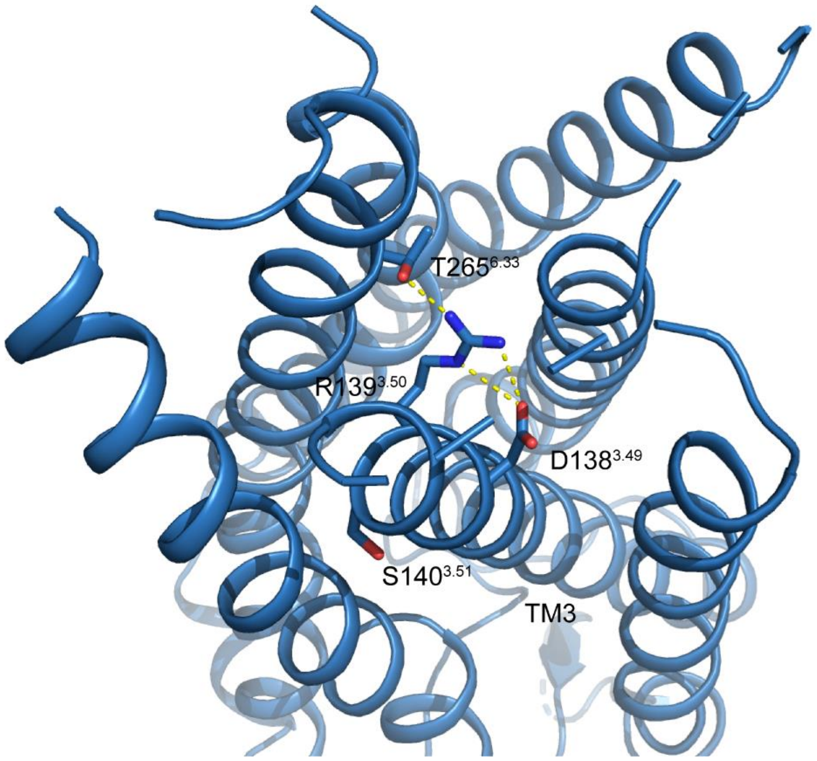

Supplementary Figure 3. $D^{3.49}$-$R^{3.50}$-$S^{3.51}$ motif in GnRH1R.

The residue R139$^{3.50}$ in $D^{3.49}$-$R^{3.50}$-$S^{3.51}$ motif of GnRH1R was locked by intra-helical hydrogen bonding with D138$^{3.49}$, as well as forming interaction with T265$^{6.33}$ in TM6. GnRH1R show as sky blue cartoon and the hydrogen bonds displayed as dotted yellow lines.

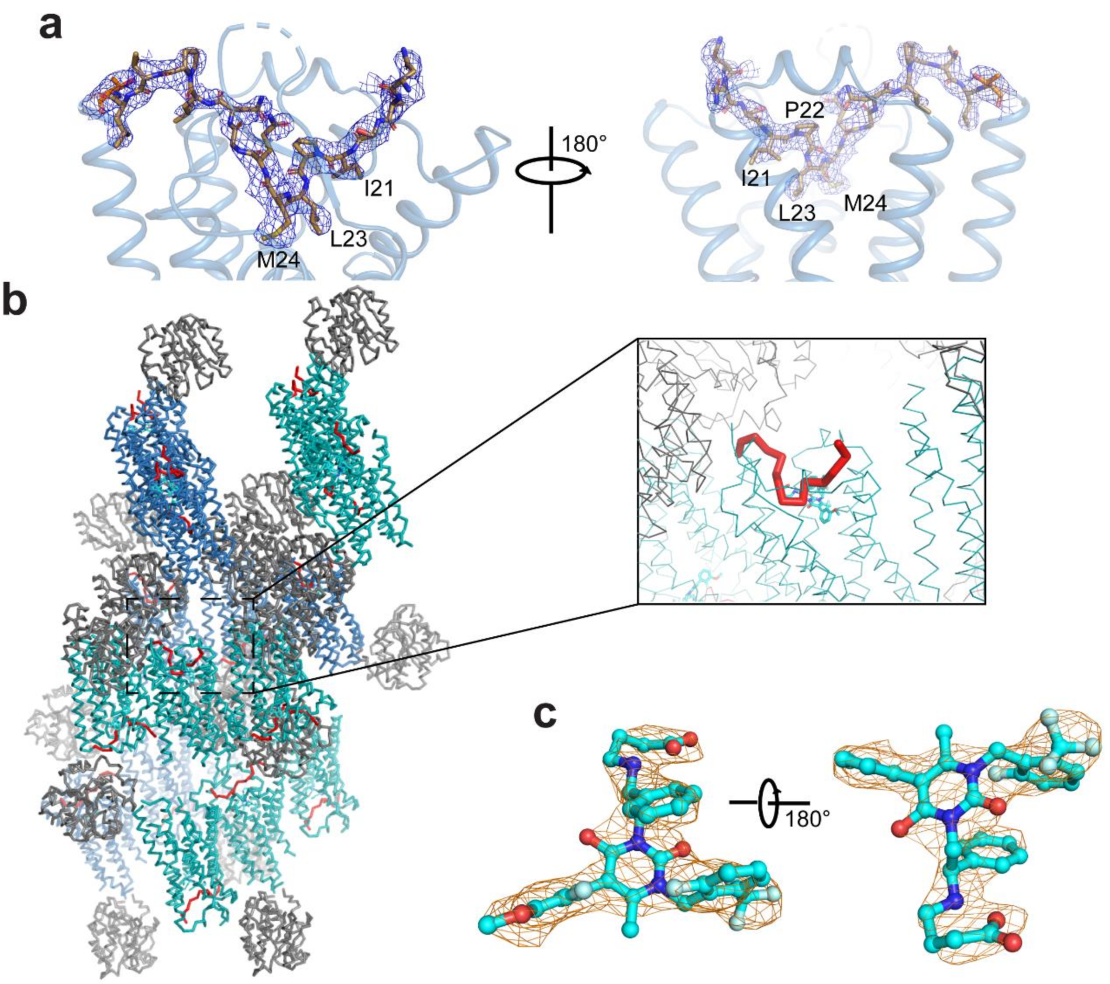

Supplementary Figure 4. Representative packing and electron density for the GnRH1R crystal structure.

(a) 2|Fo|-|Fc| map (contoured at 1.0 σ) of residues 18-33 at the N-terminal of receptor. Receptor is shown in sky blue cartoon and residues 18-33 are in sand sticks.

(b) Lattice packing interactions in the monoclinic crystals of GnRH1R (P128$^{3.39}$K)-PGS. Protomers are shown as cartoons, with the receptor component of the fusion protein colored teal or sky blue, the PGS domain was colored gray, The N terminal region of GnRH1R was shown as red ribbon.

(c) 2|Fo|-|Fc| map (contoured at 2.0 σ) for elagolix. The ligand is represented as cyan stick.

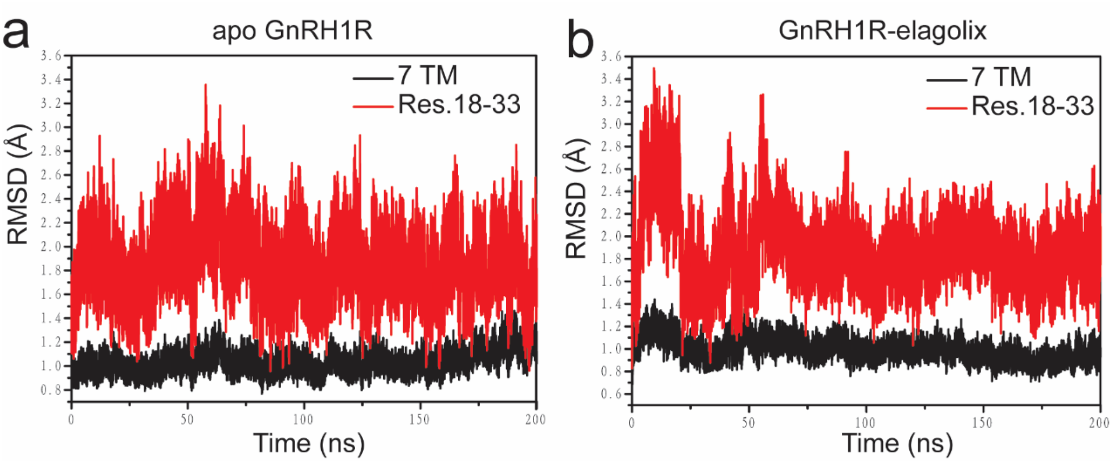

Supplementary Figure 5. Molecular dynamics (MD) simulations of the GnRH1R structure.

(a) 200-ns MD simulation of the GnRH1R apo receptor (after removing the PGS fusion protein and ligand, details were descripted in methods). Black trace was for the 7 TM of receptor, red trace was for the residues 18-33 in the N-terminal of receptor.

(b) 200-ns MD simulation of the elagolix-bound GnRH1R receptor. Black trace was for the 7TM of receptor, red trace was for the residues 18-33 in the N-terminal of the receptor. TM: transmembrane helix, Res: residues.

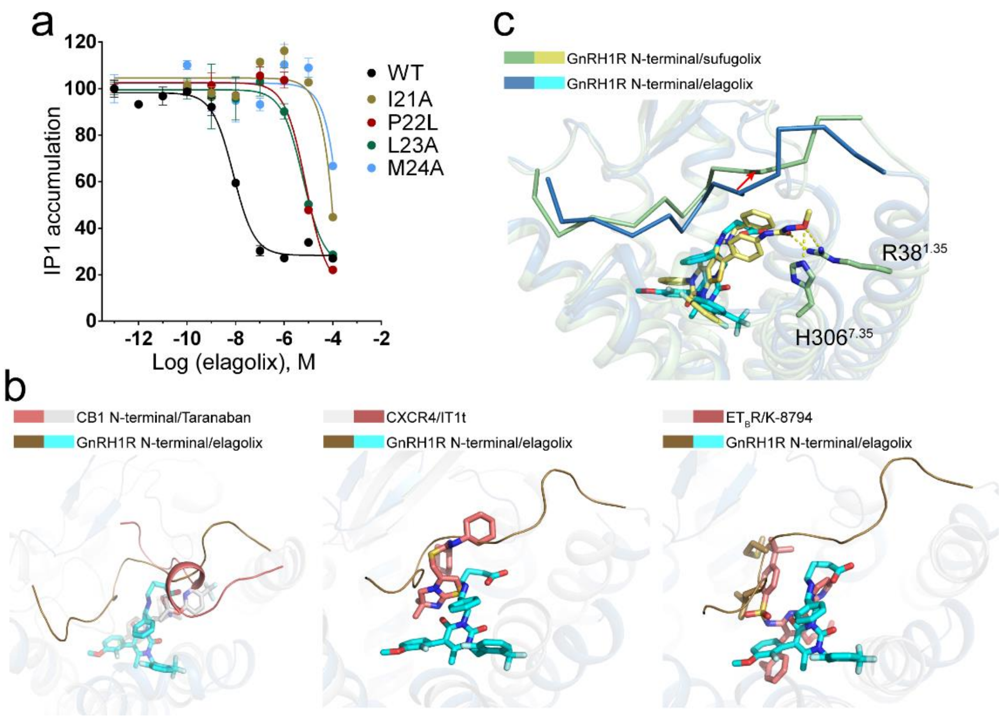

Supplementary Figure 6. Structural comparison with elagolix bound GnRH1R.

(a) Antagonist elagolix dose-dependent response curves of GnRH1R WT and N-terminal mutants stimulated by GnRH agonist determined by IP assays. HEK293T were transiently transfected with the wild-type or mutant receptors (without PGS), and IP accumulation was measured after stimulation with GnRH ligands for 2 h. IC_{50} values are expressed as means ± SEM (n=3) at 3 times independently experiment repeats with similar results. The mean EC_{50}± SEM (nM) are shown in Supplementary Table 2.

(b) Structural comparison between elagolix-bound GnRH1R with other GPCRs. Left panel: comparison with taranabant bound CB1 (PDB ID: 5U09), middle panel: comparison with T1t bound CXCR4 (PDB ID: 3ODU), right panel: comparison with K-8794 bound ET_BR (PDB ID: 5X93). N terminus of GnRH1R was shown as sand cartoon, and the ligands elagolix and was shown as cyan sticks. N terminus of CB1 was shown as pink cartoon and taranabant was shown as gray sticks. CXCR4 and ET_BR were shown as gray cartoon, IT1t and K-8794 were shown as pink sticks.

(c) Structural superposition of GnRH1R in complex with elagolix (cyan), sufugolix (pale yellow). The side-chains of $H306^{7.35}$ and $R38^{1.35}$ form hydrogen bonds with sufugolix.

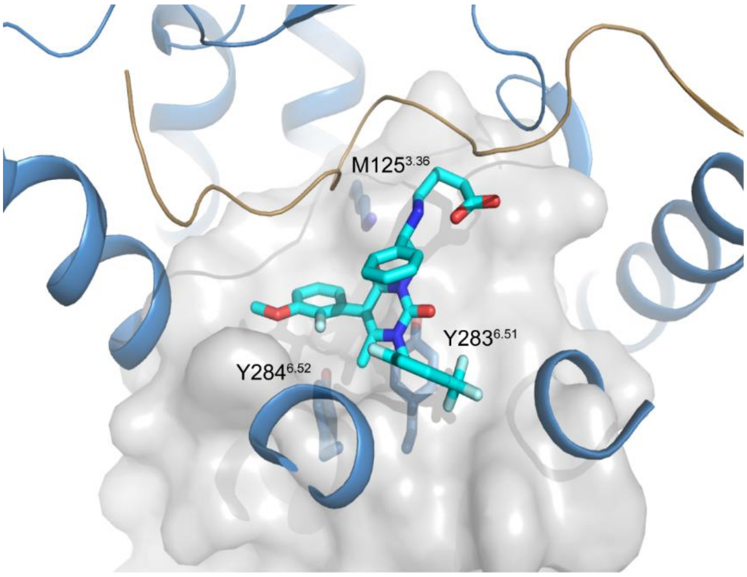

Supplementary Figure 7. The bottom wall of the orthosteric pocket in elagolix bound GnRH1R receptor.

The bottom surface is shown gray color. The side chains of M125$^{3.36}$, Y283$^{6.51}$ and Y284$^{6.52}$ are shown as sky blue sticks, and elagolix is shown as cyan sticks.

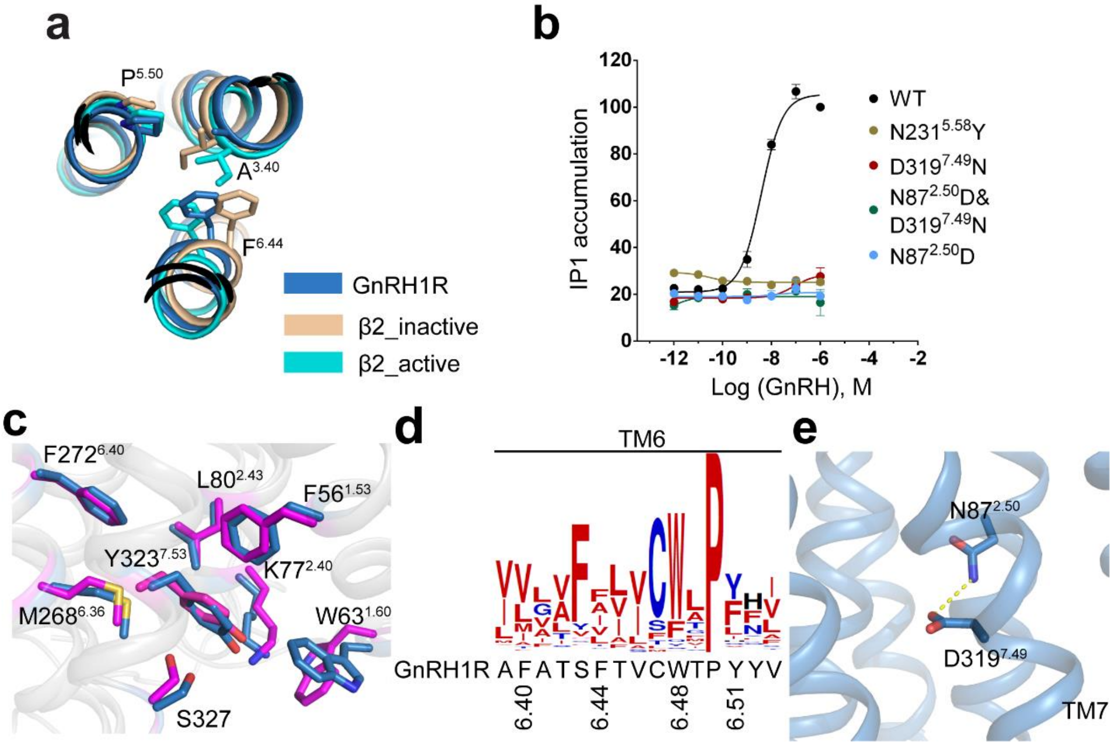

Supplementary Figure 8. Structural comparison of GnRH1R with other receptors.

(a) $P^{5.50}$-$I^{3.40}$-$F^{6.44}$ motif in both GnRH1R (sky blue) and β2 receptor, β2_inactive (PDB ID: 2RH1) displayed as wheat cartoon, β2_iactive (PDB ID: 4IDE) colored as cyan cartoon.

(b) IP accumulation assays on wild type (WT) and GnRH1R mutants. HEK293T were transiently transfected with the wild-type or mutant receptors (without PGS), and IP accumulation was measured after stimulation with GnRH ligands for 2 h. EC_{50} values are expressed as means ± SEM (n=3) at 3 times independently experiment repeats with similar results. The mean EC_{50}± SEM (nM) are shown in Supplementary Table 2.

(c) Structural comparison NPxxY motif between GnRH1R in crystal structure (sky blue) GnRH1R in 200 ns MD structure (pink).

(d) Sequence logos of TM6 for GnRH1R with class A GPCRs.

(e) The side chains of residues $N87^{2.50}$ and $D319^{7.49}$ form hydrogen bond in GnRH1R. GnRH1R show as sky blue cartoon and the hydrogen bonds displayed as dotted yellow lines.

Supplementary Table 1. Data collection and refinement statistics

	GnRH1R-PGS with elagolix[Ω]
**Data collection**	
Space group	$P3_121$
Cell dimensions	
a, *b*, *c* (Å)	74.0, 74.0, 231.2
α, β, γ (°)	90, 90, 120
Resolution (Å)	50 (2.80)[†]
R_{merge}	0.166 (1.04)
$I/\sigma I$	6.50 (0.71)
Completeness (%)	89.9 (92.4)
Redundancy	3.6
$CC_{1/2}$ in highest shell	0.53
**Refinement**	
Resolution (Å)	31.1-2.80
No. reflections	13249
R_{work} / R_{free}	0.24/ 0.28
No. atoms	
Protein	3638
Ligand	45
Other (Lipid and Solvent)	87
B-factors	
Receptor	45.3
Fusion protein	61.9
Ligand	40.0
Other (Lipid and Solvent)	50.6
R.m.s deviations	
Bond lengths (Å)	0.005
Bond angles (º)	0.78
Ramachandran plot statistics (%)	
Favored regions	93.1
Allowed regions	6.9
Disallowed regions	0

[Ω] Diffraction data from 23 crystals were merged into a complete data set.

[†] Values in parentheses are for highest-resolution shell

Supplementary Table 2.

Summary of IP assays (this study) and reported binding affinity for GnRH1R WT and mutants.

**Receptor mutant**	**Signal Transduction IP assays**				**Displacement Binding Experiments from Reference**	
		**GnRH**		**elagolix**	**GnRH**	**NBI-42902**
**GnRH1R**	**Expression level**	**EC_{50} (nM)**	**Emax (x100)**	**IC_{50} (nM)**	**IC_{50} (nM)**	**IC_{50} (nM)**
WT	1.0±0.0062	3.9±0.44	0.96	8.0±1.2	8.1[a]	0.90[a]
I21A	1.1±0.052	3.6±0.41	0.73	ND		
P22L	0.92±0.037	3.3±0.52	1.0	7.9±2.2[†]		
L23A	1.0±0.082	4.9±0.85	0.77	5.8±2.3[†]		
M24A	0.93±0.033	3.6±0.82	0.72	ND	2.2[a]	4.2[a, †]
$R38^{1.35}A$	0.84±0.023	ND	0.20	-		
$N87^{2.50}D$	0.88±0.055	ND	0.21	-		
$D98^{2.61}A$	0.94±0.021	ND	0.20	-		
$N102^{2.65}A$	1.2±0.20	0.33±0.077[†]	0.74	-		
$K121^{3.32}Q$	1.6±0.81	ND	0.21	-		
$M125^{3.36}A$	1.3±0.25	ND	0.18	-		
$P128^{3.39}K$	0.91±0.017	ND	0.24	-		
$Q174^{4.60}A$	0.88±0.13	21±2.8	0.59	-	17[b]	
$F178^{4.64}A$	0.99±0.024	76±30	0.92	-	9.3[b, †]	
$N231^{5.58}Y$	0.88±0.036	ND	0.25	-		
$F272^{6.40}V$	0.99±0.13	ND	0.28	-		
$F272^{6.40}L$	2.4±0.37	2.9±0.64	1.0	-	4.4[a]	1.5[a]
$W280^{6.48}F$	0.93±0.0040	ND	0.18	-	10[a]	5.5[a]
$Y283^{6.51}F$	1.3±0.15	7.7±3.1[†]	0.39	ND	0.32[a, †]	14[a]
$Y283^{6.51}A$	1.0±0.083	ND	19.7	-		
$Y284^{6.52}F$	2.1±0.26	7.6±1.5	0.48	1.6±0.34[†]	2.8[a]	2.3[a]
$L286^{6.54}A$	1.3±0.22	69±8.6	0.91	0.28±7.4[†]		
$Y290^{6.58}A$	0.97±0.012	2.7±0.40[†]	0.65	-		
$H306^{7.36}A$	0.91±0.020	55±25	0.29	-	51[a]	1.4[a]
$F308^{7.38}A$	1.0±0.027	4.9±0.69	1.0	35±6.5		
$D319^{7.49}N$	1.0±0.20	ND	0.28	-		
$Y323^{7.53}F$	1.3±0.20	21±5.1	0.57	-		
$Y323^{7.53}A$	0.82±0.014	ND	1.0	-		
$N87^{2.50}D$& $D319^{7.49}N$	0.95±0.012	ND	0.19	-		

ND means that the EC_{50} or IC_{50} values could not be calculated based on the experimental data, a: the dates were cited by the reference 29, b: the dates were cited by the reference 34, †: the value multiplied by 10^3. HEK293T were transiently transfected with the wild-type

or mutant receptors (without PGS), and IP accumulation was measured after stimulation with GnRH ligands for 2 h. EC_{50} values are expressed as means ± SEM (n=3) at 3 times independently experiment repeats with similar results.

Supplementary Table 3.

Summary of codon-optimized human GnRH1R gene.

codon-optimized human GnRH1 receptor gene	ATGGCCAACTCCGCCTCCCCCGAACAGAACCAGAACCACTGCTCCG<br>CTATCAACAACTCCATCCCCCTGATGCAGGGTAACCTGCCCACTCTG<br>ACCCTGAGCGGTAAGATCCGCGTCACCGTGACCTTCTTCCTGTTCCT<br>GCTGAGCGCTACCTTCAACGCTTCCTTCCTGCTGAAGCTGCAGAAGT<br>GGACCCAGAAGAAGGAAAAGGGCAAGAAGCTGTCCCGCATGAAGCT<br>GCTGCTGAAGCACCTGACTCTGGCCAACCTGCTGGAGACCCTGATC<br>GTGATGCCCCTGGACGGCATGTGGAACATCACTGTCCAGTGGTACG<br>CTGGCGAACTGCTGTGCAAGGTGCTGAGCTACCTGAAGCTGTTCAG<br>CATGTACGCTCCCGCCTTCATGATGGTGGTGATCTCCCTGGACCGCT<br>CCCTGGCTATCACTCGTCCTCTGGCCCTGAAGTCCAACTCCAAGGTG<br>GGCCAGAGCATGGTTGGCCTGGCTTGGATCCTGAGCTCCGTGTTCG<br>CTGGCCCTCAGCTGTACATCTTCCGCATGATCCACCTGGCTGACTCC<br>TCCGGTCAGACCAAGGTCTTCAGCCAGTGCGTGACTCACTGCAGCT<br>TCTCCCAGTGGTGGCACCAGGCCTTCTACAACTTCTTCACCTTCTCC<br>TGCCTGTTCATCATCCCTCTGTTCATCATGCTGATCTGCAACGCCAA<br>GATCATCTTCACCCTGACTCGCGTCCTGCACCAGGACCCCCACGAG<br>CTGCAGCTGAACCAGAGCAAGAACAACATCCCTCGCGCCCGCCTGA<br>AGACTCTGAAGATGACTGTGGCCTTCGCTACCTCCTTCACTGTCTGC<br>TGGACTCCTTACTACGTGCTGGGCATCTGGTACTGGTTCGACCCTGA<br>AATGCTGAACCGTCTGAGCGACCCTGTCAACCACTTCTTCTTCCTGT<br>TCGCCTTCCTGAACCCCTGCTTCGACCCTCTGATCTACGGTTACTTC<br>TCCCTG

Supplementary Table 4.

Primers for site-directed mutagenesis.

Primer	Forward	Reverse
pFastbac-1 vector		
P128$^{3.39}$K	AGGCCTTCATGATGGTGGTG	TAGCGTACATGCTGAACAGCTTC
pcDNA 3.1 (+) vector		
I21A	CCCCACTGATGCAGGGCAAC	CGCTGTTGTTGATGGCTGAAC
P22L	TGCTGATGCAGGGCAACCTC	GGATGCTGTTGTTGATGGCTG
L23A	CCATGCAGGGCAACCTCCC	CTGGGATGCTGTTGTTGATGGC
M24A	CCCAGGGCAACCTCCCCAC	CCAGTGGGATGCTGTTGTTGATG
R38$^{1.35}$A	CCGTGACGGTTACTTTCTTCC	CGATCTTTCCAGACAAGGTCAG
N87$^{2.50}$D	ATCTGTTGGAGACTCTGATTG	CGGCTAAGGTCAGATGTTTTAAG
D98$^{2.61}$A	CCGGGATGTGGAACATTACAG	CCAGTGGCATGACAATCAGAG
N102$^{2.65}$A	CCATTACAGTCCAATGGTATGC	CCCACATCCCATCCAGTGG
K121$^{3.32}$Q	AACTTTTCTCCATGTATGCCCC	GTAGATAACTGAGAACTTTGCAG
M125$^{3.36}$A	CCTATGCCCCAGCCTTC	CGGAGAAAAGCTTTAGATAACTG
P128$^{3.39}$K	AAGCCTTCATGATGGTGGTG	TGGCATACATGGAGAAAAGC
Q174$^{4.60}$A	GCATTATACATCTTCAGGATG	TGGTCCTGCAAAGACACTAC
F178$^{4.64}$A	GCCAGGATGATTCATCTAGC	GATGTATAACTGTGGTCCTGC
N231$^{5.58}$Y	ATGCAAAAATCATCTTCACCC	AGCAGATCAGCATGATGAAAAG
F272$^{6.40}$V	TCGCCACTTCATTTACTGTCTG	CTGCAACCGTCATTTTTAGAGTC
F272$^{6.40}$L	TAGCCACTTCATTTACTGTCTG	GTGCAACCGTCATTTTTAGAGTC
W280$^{6.48}$F	TTACTCCCTACTATGTCCTAGG	AGCAGACAGTAAATGAAGTGG
Y283$^{6.51}$F	CCTTTTATGTCCTAGGAATTTG	GAGTCCAGCAGACAGTAAATG
Y283$^{6.51}$A	CCTATGTCCTAGGAATTTGG	CGGGAGTCCAGCAGAC
Y284$^{6.52}$F	TTGTCCTAGGAATTTGGTATTG	AGTAGGGAGTCCAGCAG
L286$^{6.54}$A	CAGGAATTTGGTATTGGTTTG	CGACATAGTAGGGAGTCCAG
Y290$^{6.58}$A	CATGGTTTGATCCTGAAATG	CCCAAATTCCTAGGACATAG
H306$^{7.36}$A	CCTTCTTCTTTCTCTTTGCC	CATTTACTGGGTCTGACAAC
F308$^{7.38}$A	CCTTTCTCTTTGCCTTTTTAAAC	CGAAGTGATTTACTGGGTC
D319$^{7.49}$N	CTTTAACCCACTTATCTATGG	CATGGGTTTAAAAAGGCAAAGAG
Y323$^{7.53}$F	TTGGATATTTTTCTCTGTGATC	AGATAAGTGGATCAAAGCATG
Y323$^{7.53}$A	CCGGATATTTTTCTCTGTGATC	CGATAAGTGGATCAAAGCATG
