# Supplementary material for: Structure of the human gonadotropin-releasing hormone receptor GnRH1R reveals an unusual ligand binding mode
Source: Nat Commun. 2020 Oct 20;11:5287. doi: 10.1038/s41467-020-19109-w (PMC7576152; doi:10.1038/s41467-020-19109-w)
Supplement: Supplementary file 3 — Reporting summary [file 41467_2020_19109_MOESM3_ESM.pdf]

nature research

Corresponding author(s): Zhenhua Shao

Last updated by author(s): Sep 6, 2020

Reporting Summary

Nature Research wishes to improve the reproducibility of the work that we publish. This form provides structure for consistency and transparency in reporting. For further information on Nature Research policies, see our Editorial Policies and the Editorial Policy Checklist.

Statistics

For all statistical analyses, confirm that the following items are present in the figure legend, table legend, main text, or Methods section.

n/a	Confirmed	
☐	☒	The exact sample size (*n*) for each experimental group/condition, given as a discrete number and unit of measurement
☐	☒	A statement on whether measurements were taken from distinct samples or whether the same sample was measured repeatedly
☒	☐	The statistical test(s) used AND whether they are one- or two-sided<br>*Only common tests should be described solely by name; describe more complex techniques in the Methods section.*
☒	☐	A description of all covariates tested
☒	☐	A description of any assumptions or corrections, such as tests of normality and adjustment for multiple comparisons
☐	☒	A full description of the statistical parameters including central tendency (e.g. means) or other basic estimates (e.g. regression coefficient) AND variation (e.g. standard deviation) or associated estimates of uncertainty (e.g. confidence intervals)
☒	☐	For null hypothesis testing, the test statistic (e.g. *F*, *t*, *r*) with confidence intervals, effect sizes, degrees of freedom and *P* value noted<br>*Give P values as exact values whenever suitable.*
☒	☐	For Bayesian analysis, information on the choice of priors and Markov chain Monte Carlo settings
☒	☐	For hierarchical and complex designs, identification of the appropriate level for tests and full reporting of outcomes
☒	☐	Estimates of effect sizes (e.g. Cohen's *d*, Pearson's *r*), indicating how they were calculated

Our web collection on statistics for biologists contains articles on many of the points above.

Software and code

Policy information about availability of computer code

Data collection	X-ray diffraction data was collected at beamline 41XU at the Spring-8, Hyogo, Japan. X-ray data was indexed, integrated, and scaled using HKL3000. The GnRH1R structure was solved using Phaser in Phenix software suite (version 1.17.1-3660). The structure model was further refined in Coot (0.8.9) and Refmac5 in CCP4 package (7.0.078). Refinement parameters for elagolix ligand were generated using PRODRG web server (http://davapc1.bioch.dundee.ac.uk/prodrg/).
Data analysis	Signaling and binding assays were analyzed using Graphpad Prism 7.0. MODELLER (v9.14102), CDOCKER in the BIOVIA Discovery studio 3.1 (Accelrys Inc., San Diego, CA, USA), CHARMM-GUI web server (http://charmm-gui.org), GROMACS (2019.4) and PROPKA 3.0 simulation package were also used in this study.

For manuscripts utilizing custom algorithms or software that are central to the research but not yet described in published literature, software must be made available to editors and reviewers. We strongly encourage code deposition in a community repository (e.g. GitHub). See the Nature Research guidelines for submitting code & software for further information.

Data

Policy information about availability of data

All manuscripts must include a data availability statement. This statement should provide the following information, where applicable:

- Accession codes, unique identifiers, or web links for publicly available datasets
- A list of figures that have associated raw data
- A description of any restrictions on data availability

The PDB ID, such as 4S0V and 2BFW, used in this study are available in the Protein Data Bank (PDB). Structural data of GnRH1R has been deposited in the PDB with coordinate accession number 7BR3. Sequence of the construct used in this study is listed in Supplementary Figure 1. The source data about signaling assays are provided as a Source Data file. All other data generated or analyzed during the study are included in this article (and supplementary information files) or are

available from the corresponding author on reasonable request.

Field-specific reporting

Please select the one below that is the best fit for your research. If you are not sure, read the appropriate sections before making your selection.

- [x] Life sciences
- [ ] Behavioural & social sciences
- [ ] Ecological, evolutionary & environmental sciences

For a reference copy of the document with all sections, see nature.com/documents/nr-reporting-summary-flat.pdf

Life sciences study design

All studies must disclose on these points even when the disclosure is negative.

Sample size	In this study, the size of microcrystals sample in lipidic cubic phase are 40-50μm, so X-ray diffraction data collection for each crystal was 5-10 degree because of radiation damage. The final dataset (100% completeness) are from 23 crystals after calculation and analysis. For IP accumulation measurement and binding assay, experiments were performed as at least three independent experiments, each run at least in triplicate. The size was chosen in order to obtain an accurate S.D. or S.E. for reported values.
Data exclusions	No data was excluded from the analyses.
Replication	Protein samples were purified from different purification batch and were performed for crystallization. The procedures were repeated successfully. The final crystallography dataset are from 23 crystals, demonstrating reproducibility. The biochemical experiments in this study have been repeated by three independent experiments, and those finding are reliably reproduced.
Randomization	Randomization is not relevant to this study, as protein, crystal samples and our experiments did not involve choosing.
Blinding	Randomization is not relevant to this study, because the data collection and analysis are not biased by investigators.

Reporting for specific materials, systems and methods

We require information from authors about some types of materials, experimental systems and methods used in many studies. Here, indicate whether each material, system or method listed is relevant to your study. If you are not sure if a list item applies to your research, read the appropriate section before selecting a response.

Materials & experimental systems

n/a	Involved in the study	
x		Antibodies
	x	Eukaryotic cell lines
x		Palaeontology and archaeology
x		Animals and other organisms
x		Human research participants
x		Clinical data
x		Dual use research of concern

Methods

n/a	Involved in the study	
x		ChIP-seq
x		Flow cytometry
x		MRI-based neuroimaging

Eukaryotic cell lines

Policy information about cell lines

Cell line source(s)	Sf9 cells, Expression systems, Cat. 94011S.<br>HEK293 cells, ATCC, CRL-1573.<br>COS-7 cells, ATCC, CRL-1651.
Authentication	The cell lines are maintained by the supplier and verified by short tandem repeat (STR) profiling method. The cell used in this study are all from low-passage cell lines. Thier morphology and growth curve analysis are further checked using microscope and cell counter respectively by investigators in this study.
Mycoplasma contamination	The cell lines used in this study were negative for mycoplasma contamination.
Commonly misidentified lines (See ICLAC register)	No commonly misidentified cell lines were used.
