# Supplementary material for: Structure of the human gonadotropin-releasing hormone receptor GnRH1R reveals an unusual ligand binding mode
Source: Nat Commun. 2020 Oct 20;11:5287. doi: 10.1038/s41467-020-19109-w (PMC7576152; doi:10.1038/s41467-020-19109-w)
Supplement: Supplementary file 2 — Peer Review File [file 41467_2020_19109_MOESM2_ESM.pdf]

Editorial Note: This manuscript has been previously reviewed at another journal that is not operating a transparent peer review scheme. This document only contains reviewer comments and rebuttal letters for versions considered at *Nature Communications.*

REVIEWERS' COMMENTS

Reviewer #1 (Remarks to the Author):

The revised manuscript is significantly improved and my concerns have been addressed.

Reviewer #2 (Remarks to the Author):

the authors have sufficiently addressed the comments and suggestions that I made on the ms when submitted to another branch of the Nature family.

Reviewer #1 (Remarks to the Author):
The revised manuscript is significantly improved and my concerns have been addressed.

We thank the referee for the positive comment about our manuscript.

Reviewer #2 (Remarks to the Author):
the authors have sufficiently addressed the comments and suggestions that I made on the ms when submitted to another branch of the Nature family.

We thank the referee for the positive comment about our manuscript.
